# Prevalence of stress, anxiety and depression among healthcare workers in the Gaza strip

**DOI:** 10.1097/MD.0000000000044195

**Published:** 2025-09-05

**Authors:** Abdallah Y. Naser, Moaath Mustafa Ali, Abd Al-Karim H. Sammour, Adnan N. Skaik, Ahmed Abed, Ruba Musallam, Muath Alsarafandi, Mohammed I. Halimy, Shadi Aljerjawi, Ahmed E. Habboub, Mohamad A. Rassoul, Abdullah Ghali, Mahmoud Loubani, Ruth-Kari Ramleth, Bilal Irfan, Farashin Silevany, Majdi S. Hamarshi, Amer Hamad Issa Abukhalaf, Bashar N. Alzghoul

**Affiliations:** a Department of Applied Pharmaceutical Sciences and Clinical Pharmacy, Faculty of Pharmacy, Isra University, Amman, Jordan; b Cleveland Clinic Taussig Cancer Center, Cleveland, OH; c Islamic University of Gaza, Gaza, Palestine; d Internal Medicine, European Gaza Hospital, Gaza, Palestine; e Surgery Department, Abdali Hospital, Amman, Jordan; f Department of Orthopedic Surgery, Baylor College of Medicine, Houston, TX; g Hull University Teaching Hospitals NHS Trust, Hull, UK; h PALMED Academy, Bolton, UK; i Oslo University Hospital, Division of Mental Health and Addiction, Oslo, Norway; j NORWAC, Oslo, Norway; k Center for Bioethics, Harvard Medical School, Boston, MA; l Center for Surgery and Public Health, Brigham & Women’s Hospital, Boston, MA; m Department of Neurology, University of Michigan Medical School, Ann Arbor, MI; n Rahma Worldwide, Beverly Hills, MI; o Department of Medicine, College of Human Medicine, Michigan State University, East Lansing, MI; p Pulmonary and Critical Care Division, University of Missouri Kansas City, Kansas City, MO; q Palestinian American Medical Association, Fairfax, VA; r Nieri Department of Construction and Real Estate Development, Development and Planning, Clemson University, Clemson, SC; s Pulmonary and Critical Care Division, University of Florida, Gainesville, FL; t Pulmonary and Critical Care Medicine, Lakeland Regional Health, Lakeland, FL.

**Keywords:** anxiety, depression, Gaza strip, healthcare worker, stress, war

## Abstract

The increased workload and constant fear for life can cause significant stress and depression among healthcare workers (HCWs). The aim of this study was to measure psychological stress, depression, and their causes in HCWs who practiced in the Gaza strip since the 2023 Israel–Gaza war. We conducted a cross-sectional survey between April 2024 and January 2025 for Palestinian and international HCWs who practiced in the Gaza strip after October 7, 2023. The survey collected demographic data and measured depression using the patient health questionnaire-9 (PHQ-9), stress using the perceived stress scale (PSS-10), and anxiety using the Generalized anxiety disorder-7 (GAD-7) scale. Out of 380 HCWs who agreed to fill out the survey, 231 completed it. Most HCW from Gaza had to change their residence, work for free, and had a HCW die since war. Most international HCW from Gaza had to work for free, work for 7–30 days continuously, not take a weekly break from work, had healthcare colleagues die since war, worked in a facility that was subject to Israeli military activity, and experienced food insecurity. Most HCW (~90%) experienced severe depression, severe anxiety, and severe stress. HCWs in the Gaza Strip and on medical missions face severe working conditions, including significant workplace-related aggression and injury. The widespread depression and anxiety in HCWs in Gaza may result in long-term consequences and a psychological toll on population health.

## 1. Introduction

The 2023 Israel–Gaza war resulted in severe consequences for the Gaza strip at many levels, including demographics, architecture, agriculture, educational infrastructure, industry, transportation system, utility infrastructure, and healthcare system. By April 8, 2025, 90% of the Gaza Strip population was displaced, 50,810 fatalities and 115 thousand injuries were reported, and close to 345 thousand people were facing catastrophic levels of food insecurity.^[1]^ These numbers are believed to be underestimating the true number of traumatic injury mortality in Gaza, with one estimate concluding the number of direct deaths reached 64,260 by June 2024.^[2]^ The healthcare system in the Gaza strip is severely strained because of several factors. The military attacks on the healthcare system resulted in a significant decline in its capacity to handle the health needs of Palestinians.^[3,4]^ Around 63% of the hospitals were partially functioning, and only 19 emergency medical technicians existed for the 2.2 million population before the war. By February 24, 2025, there were 739 reported attacks on the healthcare system, which resulted in 907 fatalities, 1000 injuries, and the detention of 517 HCWs.^[5]^ The Ministry of health of the Gaza strip reported 1057 deaths among HCWs. Other factors contributing to the increased health needs are the widespread malnutrition and epidemics of infectious diseases.^[1]^ There were close to 992,000 acute respiratory infections, 575,000 acute watery diarrhea, and 107,000 cases of acute jaundice syndrome.^[6]^

The increased workload and constant fear for life can cause significant stress and depression among HCWs.^[7–10]^ Moreover, HCWs are the first to witness and treat severe injuries that result from military attacks, which can cause depression and post-traumatic stress disorder. In the study, we aim to measure psychological stress, depression, and their causes in HCWs who practiced in the Gaza strip since the 2023 Israel–Gaza war.

## 2. Methods

### 2.1. Study design, setting, and participants

We conducted an anonymous cross-sectional online survey to measure stress, anxiety, and depression prevalence among HCWs. Eligibility criteria included any HCW who practiced in the Gaza strip after October 7, 2023. HCWs included nurses, physicians, physician assistants, nurse practitioners, pharmacists, emergency medical technicians/paramedics, and others. The study included Palestinian HCWs in Gaza and international HCWs arriving on medical mission trips. Healthcare students, including medical and nursing students, were excluded.

### 2.2. Survey design and distribution

The survey questions are provided in the supplementary file, Supplemental Digital Content, https://links.lww.com/MD/P790. The survey included questions about age, sex, profession type, physicians’ subspecialty, and primary residence before the war. Moreover, the survey included questions about whether respondents were: volunteering and reason, displaced, making an income, ability to take a break from work, the demise of a healthcare colleague, injury of healthcare colleague, personal injury, experiencing a military raid to healthcare facility while present, injury or death of a patient during a military raid to a healthcare facility while present, weight loss, demise or injury of immediate or distant relatives, personal history of chronic disease and access to medications for personal illness. It also inquired about the longest stretch of working days (shifts), the longest stretch of working hours per shift, and sleeping patterns. Lastly, the survey included questions screening for depression using the patient health questionnaire-9 (PHQ-9), stress using the perceived stress scale (PSS-10), and anxiety using the generalized anxiety disorder-7 (GAD-7) scale.^[11–13]^

The survey was distributed in both English and Arabic versions. The English version was distributed mainly to international HCWs who practiced in Gaza on medical missions, and the Arabic version was mainly distributed to HCWs indigenous to the Gaza strip. The English version was translated into Arabic by an Arabic and English-speaking physician and confirmed for accuracy by another investigator. The survey was created and distributed using a cloud-based platform, Qualtrics.^[14]^ Responses were stored securely in the Qualtrics database.^[14]^ The message used for disseminating the questionnaire is provided in the supplemental file, Supplemental Digital Content, https://links.lww.com/MD/P790.

Initially, the survey was piloted on 10 HCWs to check for errors and the clarity of questions. It was then distributed from April 2024 till January 2025, with a sample size goal of 350 respondents. It was distributed using 2 mechanisms: On the ground by a medical team inside the Gaza strip, where the respondents were asked to fill out the survey, By emails and text messages distributed by team leaders of medical missions to healthcare providers. The initial goal was to complete the sample size by July 2024. However, because of slow recruitment, intermittent internet availability, displacement of the research team, and severe war conditions, the study was delayed.

### 2.3. Sample size determination

We used the following equation for sample size estimation: sample size = (*z*^2^ × *p* (1−*p*)/*e*^2^)/ (1 + ((*z*^2^ × *p* (1−*p*))/*e*^2^ *N*)), where *N* is the population size, *e* is the margin of error and *z* is the *z*-score. There are no accurate estimates of the size of HCWs currently in the Gaza strip, especially after the war was active for several months. Moreover, we were not able to determine the number of HCWs who would enter Gaza on medical missions. The number of doctors per 1 thousand people in the West Bank and the Gaza strip is estimated to be around 2.2 physicians per 1000 population size. Using that estimate, the population size of doctors in Gaza would be close to 4840 before the war.^[15]^ The number of nurses per 1 thousand people in the West Bank and Gaza is estimated to be around 2 nurses per 1000 population size. Using that estimate, the population size of nurses in Gaza would be close to 4400.^[16]^ Using physician and nurse population sizes of 4840 and 4400, a confidence interval of 95%, and a margin of error of 5%, the sample size is estimated to be 356 and 354 responses, respectively. Using a HCW population size of 30,000, a confidence interval of 95%, and a margin of error of 5%, the sample size is estimated to be 380 responses. Despite the initial goal to collect more than 380 responses, we concluded the study when we had more than 350 responses, given the difficulty in collecting responses, and the final date of collection was the end of January 2025.

### 2.4. Statistical analysis

Descriptive statistics were used to summarize variables. Continuous data was summarized as medians with interquartile ranges or averages with standard deviations. Categorical variables were presented as absolute numbers and summarized as percentages. Linear regression was used to determine the association between baseline factors with depression, anxiety and stress. All statistical tests were 2-sided, and *P*-values < .05 were considered statistically significant. Statistical package for the social sciences (SPSS), version 29, statistical software was used for analysis.

## 3. Results

### 3.1. Respondent characteristics

Between April 2024 and January 2025, 380 HCWs opened the survey, and 231 completed it. Fifty-eight participants filled out the English version, and 173 filled out the Arabic version. Table 1 summarizes the demographic characteristics of HCW participants from inside and outside Gaza. Among participants in Gaza (*n* = 190), most were between the ages of 24 and 30 (*n* = 96, 50.5%), and two-thirds were males. Almost half of the participants were physicians, and 18% were nurses. Among the physicians, general practitioners (*n* = 45, 43.3%) and orthopedics (*n* = 20, 19.2%) were the 2 most common specialties. Among international HCW participants (*n* = 36), 60% of participants were 41 years or older, and two-thirds were males. Two-thirds were physicians, and almost one-third of the physicians specialized in orthopedics (*n* = 7, 30.4%).

**Table 1:** Baseline demographics of participants in healthcare workers surveyed in Gaza post-October 2023.

Variable	Inside Gaza* (*n* = 190)		Outside Gaza (*n* = 36)	
	Frequency	Percentage, %	Frequency	Percentage, %
Sex				
Female	58	30.5	13	36.1
Male	132	69.5	23	63.9
Age groups				
18–23 yr	33	17.4	4	11.1
24–30 yr	96	50.5	6	16.7
31–40 yr	33	17.4	5	13.9
41–50 yr	21	11.1	7	19.4
51–60 yr	5	2.6	7	19.4
61 yr and above	2	1.0	7	19.4
Profession				
Nurse	35	18.4	6	16.7
Community healthcare workers	1	0.5	1	2.8
Doctor (i.e., physician)	104	54.7	23	63.9
Support staff (janitor, food service staff, administrators, etc)	4	2.1	0	0.0
EMT/paramedic/emergency medicine	12	6.3	1	4.3
Physician assistant or nurse practitioner	4	2.1	1	2.8
Pharmacist	7	3.7	1	2.8
Other areas of patient care (laboratory technician, x-ray, therapist, front desk, etc)	17	8.9	1	2.8
Others	6	3.2	2	5.6
Physician specialty				
General practitioner	45	43.3	1	4.3
General surgery	5	4.8	2	8.7
Pediatrics	1	1.0	3	13.0
Internal medicine (general)	3	2.9	2	5.6
Cardiology	1	1.0	0	0.0
Urology or nephrology	1	1.0	1	4.3
Hematology	1	1.0	0	0.0
Orthopedics	20	19.2	7	30.4
Dentistry	1	1.0	0	0.0
Specialty surgeon (e.g., ophthalmology, otolaryngology, etc)	6	5.8	2	8.7
Radiologist	14	13.5	0	0.0
Another specialty	2	1.9	2	8.7
Anesthesiology	4	3.8	3	13.0
Primary residence before October 2023				
Bani Suheila	10	5.3	0	0.0
Beit Hanoun	7	3.7		
Beit Lahiya	17	8.9		
Deir Al-Balah	23	12.1		
Gaza City	69	36.3		
Jabalia	14	7.4		
Khan Yunis	30	15.8		
Rafah	20	10.5		

* Data for this item were missing for 5 participants.

Among international HCWs (*n* = 36), 26 stated they had volunteered to work in Gaza after October 2023 (72.2%). Of those who volunteered, 8 (30.7%) started at the beginning of the war, 0 (0.0%) started a month ago, 2 (7.6%) started 2 months ago, and 14 (53.8%) started 3 months ago or more. Among them, 25 responded that they would volunteer again in the future if possible (96.2%). Regarding the reasons for volunteering, humanitarian motivation was the most reported reason for volunteering (*n* = 26, 72.2%), followed by religious (*n* = 11, 30.6%) and career motivation (*n* = 4, 11.1%), with a few reporting other reasons (*n* = 3, 8.3%).

Among the international HCWs, the majority were from the USA (20, 57.1%), followed by Norway (9, 25.7%). Two healthcare providers from the UK (5.7%), while 1 healthcare provider came from each of the following: Amman, Canada, Germany and other European countries (2.9% each). Two participants did not report on their country of residence.

### 3.2. Impact of war on participants

The impact of war on participants is summarized in Table 2. Most HCWs inside the Gaza strip had to relocate, work in a field hospital, work for free, had a colleague who died or injured after October 2023, personally being injured, had a patient who they treated die from military act (directly or indirectly), experienced food insecurity, lost some weight, lost their home, lost immediate family and were unable to secure medications for self-illnesses.

**Table 2:** Impact of war on participants in healthcare workers surveyed in Gaza post-October 2023.

	Inside Gaza (*n* = 190)		Outside Gaza (*n* = 36)	
	*n*	%	*n*	%
Number of times participant relocated since October 7, 2023				
Once	0	0.0%	5	13.9%
2–3 times	8	4.2%	0	0.0%
>3 times	10	5.3%	1	2.8%
No, I did not move	7	3.7%	18	50.0%
Did not answer	165	86.8%	12	33.3%
Current location of work				
Established hospital (governmental)	33	17.4%	10	27.8%
Outpatient clinic	32	16.8%	3	8.3%
Field hospital	110	57.9%	6	16.7%
Blood bank	10	5.3%	2	5.6%
Dialysis center, separate from hospital	0	0.0%	0	0.0%
Somewhere else	5	2.6%	15	41.7%
Did not answer	0	0.0%	0	0.0%
Need to transfer job from one location to another as a healthcare provider since October 7, 2023?				
No	9	4.7%	19	52.8%
Yes	16	8.4%	5	13.9%
Did not answer	165	86.8%	12	33.3%
Number of times the healthcare provider had to transfer your job from one location to another as a healthcare provider since October 7, 2023				
No, I did not have to change my job location	83	43.7%	29	80.6%
1 time	92	48.4%	4	11.1%
2–3 times	14	7.4%	1	2.8%
>3 times	1	0.5%	2	5.6%
Did not answer	0	0.0%	0	0.0%
Making an income from the healthcare service you are providing in Gaza				
No	127	66.8%	34	94.4%
Yes	63	33.1%	2	5.6%
Did not answer	0	0.0%	0	0.0%
Longest continuous period of work (i.e., working shift) since October 7, 2023				
<1 wk	107	56.3%	10	27.8%
7–30 d	65	34.2%	23	63.9%
31–60 d	1	0.5%	3	8.3%
61–90 d	1	0.5%	0	0.0%
91–120 d	0	0.0%	0	0.0%
>120 d	16	8.4%	0	0.0%
Did not answer	0	0.0%	0	0.0%
Average number of hours of sleep the healthcare worker had per night over the past month				
<2 h	14	7.4%	2	5.6%
2–4 h	15	7.9%	4	11.1%
4–6 h	27	14.2%	9	25.0%
6–8 h	22	11.6%	13	36.1%
>8 h	9	4.7%	1	2.8%
Did not answer	103	54.2%	7	19.4%
Take a break from work every day				
No	80	42.1%	8	22.2%
Yes	105	55.3%	28	77.8%
Did not answer	5	2.6%	0	0.0%
How long is the break approximately?				
2 h	98	51.6%	16	44.4%
4 h	72	37.9%	8	22.2%
6 h	3	1.6%	2	5.6%
8 h	4	2.1%	0	0.0%
10 h	0	0.0%	2	5.6%
>10 h	0	0.0%	4	11.1%
Did not answer	13	6.8	4	11.1
Take a break from work every week				
No	45	23.7%	18	50.0%
Yes	36	18.9%	12	33.4%
Did not answer	109	57.4%	6	16.6%
If yes, how long?				
<1 d	49	25.8%	5	13.9%
1 d	127	66.8%	10	27.8%
2 d	5	2.6%	4	11.1%
>2 d	0	0.0%	3	8.3%
Did not answer	9	4.7%	14	38.9%
Healthcare colleagues the participant work(ed) with died because of military action since October 7, 2023				
No	54	28.4%	13	36.1%
Yes	88	46.3%	19	52.8%
Did not answer	48	25.3%	4	11.1%
A healthcare colleagues the participant work(ed) with been injured because of military action since October 7, 2023				
No	28	14.7%	13	36.1%
Yes	162	85.3%	23	63.9%
Did not answer	0	0.0%	0	0.0%
Participant has been directly or indirectly targeted by military action since October 7, 2023				
No	35	18.4%	21	58.3%
Yes	155	81.6%	15	41.7%
Did not answer	0	0.0%	0	0.0%
Participant has been directly or indirectly targeted by military action since October 7, 2023, and was injured				
No	81	42.6%	10	27.8%
Yes	96	50.5%	9	25.0%
Did not answer	13	6.8%	17	47.2%
Participant has been directly or indirectly targeted by military action since October 7, 2023, and had an amputation				
No	78	41.1%	6	16.7%
Yes	18	9.5%	3	8.3%
Did not answer	94	49.5%	27	75.0%
The amputation happened due to injury while working				
No	24	12.6%	4	11.1%
Yes	4	2.1%	6	16.7%
Did not answer	162	85.3%	26	72.2%
The medical facilities where participant worked was involved in a military action				
No	78	41.1%	20	55.6%
Yes	40	21.1%	13	36.1%
Did not answer	72	62.1%	3	8.3%
If yes, any patient was injured				
No	4	10.0%	2	15.4%
Yes	36	90.0%	11	84.6%
Did not answer	0	0.0%	0	0.0%
Patients the participant were caring for die directly or indirectly from military actions				
No	14	7.4%	5	13.9%
Yes	119	62.6%	12	33.3%
Did not answer	57	30.0%	19	52.8%
Opinion on healthcare community outside Gaza whether they have done enough to provide medical care in Gaza				
No	41	21.6%	22	61.1%
Yes	91	47.9%	8	22.2%
Did not answer	58	30.5%	6	16.7%
Food insecure				
No	138	72.6%	32	88.9%
Yes	52	27.4%	4	11.1%
Did not answer	0	0.0%	0	0.0%
Weight have you lost in kilograms since October 7, 2023				
I did not lose weight.	17	8.9%	3	8.3%
<5 kg	150	78.9%	12	33.3%
6–10 kg	5	2.6%	0	0.0%
11–15 kg	5	2.6%	0	0.0%
>15 kg	5	2.6%	0	0.0%
I have lost weight, but I am not sure what weight I have lost	0	0.0%	0	0.0%
Did not answer	8	4.2%	21	58.3%
Losing primary home (i.e., partially or completely destroyed) since October 7, 2023				
No	64	33.7%	28	77.8%
Yes	95	50.0%	6	16.7%
Did not answer	31	16.3	2	5.6%
Loss of immediate family members (father, mother, brother, sisters, wife, or children) (i.e., died) since October 7, 2023				
No	48	25.3%	29	80.6%
Yes	142	74.7%	7	19.4%
Did not answer	0	0.0%	0	0.0%
Number of immediate family members (father, mother, brother, sisters, wife, or children) have died/ been martyred				
0	107	75.4%	5	71.4%
1–2	33	23.2%	2	28.6%
3–4	1	0.7%	0	0.0%
5–6	0	0.0%	0	0.0%
7–8	1	0.7%	0	0.0%
>8	0	0.0%	0	0.0%
Did not answer	0	0.0%	0	0.0%
Loss of any of participant’s indirect family members (i.e., died) since October 7, 2023				
No	27	14.2%	23	63.9%
Yes	21	11.0%	3	8.3%
Did not answer	142	74.8%	10	27.8%
Number of indirect family members died/been martyred				
0	0	0.0%	1	33.3%
1–2	4	20.0%	1	33.3%
3–4	2	10.0%	1	33.3%
5–6	3	15.0%	0	0.0%
7–8	1	5%	0	0.0%
>8	10	50%	0	0.0%
Did not answer	0	0.0%	0	0.0%
Immediate family members who have been directly or indirectly targeted by military action since October 7, 2023				
No	58	30.5%	25	69.5%
Yes	41	21.5%	3	8.4%
Did not answer	91	48.0%	8	22.3%
If yes, anyone been injured				
No	122	64.2%	11	30.6%
Yes	52	27.4%	3	8.3%
Did not answer	16	8.4%	22	61.1%
If yes, any amputations been performed				
No	14	7.4%	0	0.0%
Yes	38	20.0%	3	8.3%
Did not answer	138	72.6%	33	91.7%
If yes, anyone died				
No	21	11.1%	1	2.8%
Yes	16	8.4%	2	5.6%
Did not answer	153	80.5%	33	91.7%
Chronic medical disease(s) in participants				
No	21	11.1%	16	44.0%
Yes	17	8.9%	9	25.0%
Did not answer	152	80.0%	11	30.6%
Ability to secure medications for personal diseases				
No	142	74.7%	13	36.1%
Yes	26	13.7%	7	19.4%
Did not answer	22	11.6%	16	44.4%
Ability to receive health care for personal diseases				
No	16	8.4%	2	5.6%
Yes	14	7.4%	7	19.4%
Did not answer	160	84.2%	27	75.0%
Personal difficulty to do job, take care of household chores, or getting along with other people				
There is no difficulty	28	14.7%	12	33.3%
There are some difficulties	65	34.2%	16	44.4%
There are severe difficulties	29	15.3%	4	11.1%
There are very complex difficulties	68	35.8%	4	11.1%
Did not answer	0	0.0%	0	0.0%

For international HCWs, 50% had to relocate their work during their medical missions. Most had to work for free, work for 7 to 30 days continuously, not take a weekly break from work, had healthcare colleagues die after October 2023, worked in a facility that was involved in a military act, and experienced food insecurity. Around 60% of them believed that the healthcare community outside Gaza has not done enough to provide medical care in Gaza.

### 3.3. Depression, anxiety, and stress in healthcare workers

Table 3 summarizes the frequency of depression symptoms and anxiety among HCWs who worked in Gaza after October 2023 using the PHQ-9 and the GAD-7 scale. Among the participants, severe depression was most prevalent, with 217 individuals (93.9%) scoring between 20 and 27 on the PHQ scale. Additionally, 12 participants (5.2%) experienced moderately severe depression, while 2 participants (0.9%) reported moderate depression. In terms of anxiety, the majority also exhibited severe anxiety levels, with 223 individuals (96.5%) affected. Only 7 participants (1.2%) were found to have minimal anxiety levels.

**Table 3:** Frequency of depression and anxiety symptoms among healthcare workers who worked in Gaza after October 2023 using the patient health questionnaire-9 (PHQ-9) and the Generalized anxiety disorder-7 (GAD-7) scale.

Over the past 2 weeks, how many times have you experienced any of the following problems?	Not at all	Several days	More than half the days	Nearly every day	Not answered
Patient health questionnaire-9 (PHQ-9)					
Little interest or pleasure in doing things?	19 (25.0%)	37 (48.7%)	9 (11.8%)	11 (14.5%)	155 (67.1%)
Feeling down, depressed, or hopeless?	30 (13.2%)	81 (35.7%)	38 (16.7%)	78 (34.4%)	4 (1.7%)
Trouble falling or staying asleep, or sleeping too much?	22 (9.7%)	91 (40.1%)	32 (14.1%)	82 (36.1%)	4 (1.7%)
Feeling tired or having little energy?	15 (6.6%)	96 (42.3%)	47 (20.7%)	69 (30.4%)	4 (1.7%)
Poor appetite or overeating?	27 (11.9%)	73 (32.2%)	46 (20.3%)	81 (35.7%)	4 (1.7%)
Feeling bad about yourself – or that you are a failure or have let yourself or your family down?	34 (15.0%)	78 (34.4%)	46 (20.7%)	69 (30.4%)	4 (1.7%)
Trouble concentrating on things, such as reading the newspaper or watching television?	57 (25.2%)	80 (35.4%)	35 (15.5%)	54 (23.9%)	5 (2.2%)
Moving or speaking so slowly that other people could have noticed? Or the opposite – being so fidgety or restless that you have been moving around a lot more than usual?	46 (20.4%)	73 (32.3%)	43 (19.0%)	64 (28.3%)	5 (2.2%)
Thoughts that you would be better off dead, or of hurting yourself in some way?	91 (40.3%)	69 (30.5%)	28 (12.4%)	38 (16.8%)	5 (2.2%)
Generalized anxiety disorder-7 (GAD-7)					
Feeling nervous, anxious, or on edge	93 (41.2%)	53 (23.5%)	29 (12.8%)	51 (22.6%)	5 (2.2%)
Not being able to stop or control worrying	23 (10.2%)	84 (37.2%)	50 (22.1%)	69 (30.5%)	5 (2.2%)
Worrying too much about different things	41 (18.1%)	86 (38.1%)	40 (17.7%)	59 (26.1%)	5 (2.2%)
Trouble relaxing	45 (19.9%)	85 (37.6%)	42 (18.6%)	54 (23.9%)	5 (2.2%)
Being so restless that it’s hard to sit still	28 (12.4%)	97 (42.9%)	48 (21.2%)	53 (23.5%)	5 (2.2%)
Becoming easily annoyed or irritable	40 (17.7%)	91 (40.3%)	51 (22.6%)	44 (19.5%)	5 (2.2%)
Feeling afraid as if something awful might happen	35 (15.5%)	85 (37.6%)	41 (18.1%)	65 (28.8%)	5 (2.2%)

Table 4 summarizes the frequency of stress symptoms among HCWs who worked in Gaza after October 2023 using the PSS-10 scoring system. Only 3 participants (1.3%) experienced a low level of stress, while 21 participants (9.1%) reported a moderate stress level. Most participants (202, 87.4%) experienced a high level of stress. Five participants did not answer (2.2%).

**Table 4:** Frequency of symptoms of stress over last month among healthcare workers who worked in Gaza after October 2023 using the PSS-10 scale.

The following questions inquire about your feelings and thoughts over the past month. Please answer all the questions.	Never	Almost never	Sometimes	Fairly often	Very often	Not answered
How often have you been upset because of something that happened unexpectedly?	10 (4.3%)	114 (49.4%)	76 (32.9%)	17 (7.4%)	9 (3.9%)	5 (2.2%)
How often have you felt that you were unable to control the important things in your life?	9 (3.9%)	32 (13.9%)	76 (32.9%)	75 (32.5%)	33 (14.3%)	6 (2.6%)
How often have you felt nervous and stressed?	6 (2.6%)	42 (18.2%)	95 (41.1%)	60 (26.0%)	22 (9.5%)	6 (2.6%)
How often have you felt confident about your ability to handle your personal problems?	10 (4.3%)	19 (8.2%)	69 (29.9%)	78 (33.8%)	48 (20.8%)	7 (3.0%)
How often have you felt that things were going your way?	2 (0.9%)	36 (15.6%)	89 (38.5%)	69 (29.9%)	28 (12.1%)	7 (3.0%)
How often have you found that you could not cope with all the things that you had to do?	18 (7.8%)	67 (29.0%)	86 (37.2%)	35 (15.2%)	17 (7.4%)	8 (3.5%)
How often have you been able to control irritations in your life?	4 (1.7%)	43 (18.6%)	89 (38.5%)	69 (29.9%)	19 (8.2%)	7 (3.0%)
How often have you felt that you were on top of things?	4 (1.7%)	58 (25.1%)	93 (40.3%)	50 (21.6%)	18 (7.8%)	8 (3.5%)
How often have you been angered because of things that happened that were outside of your control?	13 (5.6%)	46 (19.9%)	98 (42.4%)	47 (20.3%)	19 (8.2%)	8 (3.5%)
How often have you felt difficulties were piling up so high that you could not overcome them?	10 (4.3%)	31 (13.4%)	79 (34.2%)	82 (35.5%)	22 (9.5%)	7 (3.0%)

In Table 5 and Tables S1 to S3 (Supplemental Digital Content, https://links.lww.com/MD/P791), we summarized the associations between the baseline characteristics of participants and symptoms of depression, anxiety, and perceived stress. We found that the depression total score was higher in individuals with anxiety (*P* < .05) and obstetrics and gynecology physicians (*P* < .05). Regarding anxiety, it was higher in patients with high stress scores and high depression scores (*P* < .05). Lastly, regarding perceived stress, it was higher in individuals who had high anxiety scores and urology/ nephrology physicians (*P* < .05).

**Table 5:** Depression, anxiety and perceived stress scores across demographic characteristics of the participants.

Variable	Depression	*P* value	Anxiety	*P* value	Perceived stress	*P* values
Gender						
Female	41.00 ± 11.72	.03	32.10 ± 5.99	.08	32.10 ± 5.99	.27
Male	37.48 ± 11.91		31.20 ± 5.58		31.20 ± 5.58	
Age						
18–23 yr	41.38 ± 10.94	.001	32.78 ± 7.97	.30	32.78 ± 7.97	.67
24–30 yr	39.04 ± 10.77		31.20 ± 5.07		31.20 ± 5.07	
31–40 yr	41.53 ± 13.36		31.50 ± 4.96		31.50 ± 4.96	
41–50 yr	36.32 ± 12.52		30.96 ± 6.16		30.96 ± 6.16	
51–60 yr	32.58 ± 13.20		32.42 ± 4.03		32.42 ± 4.03	
61 yr and above	23.50 ± 5.78		29.25 ± 5.47		29.25 ± 5.47	
Occupation						
Nurse	38.29 ± 11.74	.84	29.93 ± 6.90	0.27	29.93 ± 6.90	.25
Community healthcare workers	42.00 ± 15.56		25.00 ± 18.38		25.00 ± 18.38	
Doctor (i.e., physician)	37.87 ± 12.53		31.59 ± 5.27		31.59 ± 5.27	
Support staff (janitor, food service staff, administrators, etc)	48.50 ± 10.08		32.50 ± 2.38		32.50 ± 2.38	
EMT/paramedic	41.00 ± 0.00		37.00 ± 0.00		37.00 ± 0.00	
Physician assistant or nurse practitioner	44.40 ± 13.94		34.20 ± 3.35		34.20 ± 3.35	
Pharmacist	37.38 ± 6.52		31.00 ± 3.89		31.00 ± 3.89	
Other areas of patient care (laboratory technician, x-ray, therapist, front desk, etc)	40.44 ± 11.90		33.56 ± 6.40		33.56 ± 6.40	
Others	37.13 ± 11.13		30.25 ± 2.71		30.25 ± 2.71	
Emergency medicine	39.58 ± 10.06		32.83 ± 5.32		32.83 ± 5.32	
Subspeciality						
General practitioner	37.65 ± 9.70	.70	30.78 ± 3.72	.52	30.78 ± 3.72	.24
General surgery	36.00 ± 17.45		32.86 ± 6.20		32.86 ± 6.20	
Pediatrics	35.00 ± 14.00		32.50 ± 3.32		32.50 ± 3.32	
Internal medicine (general)	44.60 ± 14.64		28.00 ± 10.65		28.00 ± 10.65	
Cardiology	48.00 ± 0.00		31.00 ± 0.00		31.00 ± 0.00	
Urology or nephrology	25.50 ± 6.36		31.50 ± 3.54		31.50 ± 3.54	
Hematology	56.00 ± 0.00		44.00 ± 0.00		44.00 ± 0.00	
Orthopedics	39.56 ± 12.64		32.48 ± 6.15		32.48 ± 6.15	
Dentistry	26.00 ± 0.00		27.00 ± 0.00		27.00 ± 0.00	
Specialty surgeon (e.g., ophthalmology, otolaryngology …etc)	37.50 ± 12.59		31.88 ± 4.22		31.88 ± 4.22	
Radiologist	37.07 ± 14.37		30.36 ± 4.31		30.36 ± 4.31	
Another specialty	38.50 ± 18.57		32.25 ± 5.74		32.25 ± 5.74	
Anesthesiology	34.43 ± 17.92		35.00 ± 6.90		35.00 ± 6.90	
Residence						
Bani Suheila	39.80 ± 10.03	.01	28.20 ± 10.09	.01	28.20 ± 10.09	.01
Beit Hanoun	41.43 ± 10.98		30.14 ± 5.01		30.14 ± 5.01	
Beit Lahiya	41.41 ± 14.42		33.76 ± 6.30		33.76 ± 6.30	
Deir Al-Balah	39.39 ± 11.14		30.83 ± 2.41		30.83 ± 2.41	
Gaza city	40.13 ± 10.20		32.35 ± 4.87		32.35 ± 4.87	
Jabalia	44.86 ± 10.41		34.29 ± 6.02		34.29 ± 6.02	
Khan Yunis	38.87 ± 14.00		32.10 ± 5.09		32.10 ± 5.09	
Rafah	39.10 ± 13.23		30.95 ± 4.71		30.95 ± 4.71	
Another area, including outside the Gaza strip (mention it)	29.92 ± 9.63		29.03 ± 6.84		29.03 ± 6.84	

## 4. Discussion

In this study, we aimed to measure the psychological impact of the war on HCWs and associated factors. We found that depression, anxiety, and perceived stress were pervasive and severe among HCWs from Gaza. We surveyed several factors that are known to be associated with psychological illness, including loss of residence, demise of family members, long working hours, displacement, demise of patients, and not being able to self-care. We found that most HCWs in Gaza experienced several of these factors.

There is limited research on psychological stress among HCWs.^[17]^ In a study by Hakhmigari and Diamant, they surveyed 374 nonmilitary employees in Israel after the October 7^th^, 2023 war and found that 1.1% were physically injured and 17.1% reported physical injury of friends or relatives.^[18]^ The study also confirmed increased burnout among the participants. In contrast, our study included only HCWs and found a much higher incidence of personal injury and family injury. In another study in Israel, hospital personnel who worked in hospitals exposed to war-related stress were more likely to manifest post-traumatic stress disorder and depression.^[19]^ HCWs in Gaza experienced severe work-related conditions, and many were personally injured or persecuted. In another study by Elhadi, they found increased emotional exhaustion, reported depersonalization, and a lower sense of personal accomplishment among 532 Libyan HCWs during COVID-19 and civil war.^[20]^ Similarly, the Ukraine-Russia war was associated with increased burnout among HCWs.^[7,21]^ The psychological impact of the Gaza war on young Palestinian medical students in Gaza has been examined by Aldabbour and his colleagues. Similar to our study, they found that a majority had lost their homes and income, developed mild depressive symptoms or higher, and reported mild anxiety and mild stress symptoms or higher.^[22]^

Our study found that a significant number of HCWs in Gaza faced safety issues and were personally harmed. Our findings are consistent with reports from the UN and other sources, including the Ministry of Health in the Gaza strip.^[5]^ A study by Asi and her group showed that close to 68% of healthcare facilities have sustained damage, multi-temporal coherent change detection on Copernicus sentinel 1-A synthetic aperture radar imagery.^[4]^ Other studies found similar findings.^[3]^ HCWs are supposed to be protected during armed conflict under several international legal frameworks, like the Geneva convention I articles 24, 25, and 28, the Geneva convention V articles 18 and 20, and their additional protocols.^[23]^ Moreover, the Rome statute of the international criminal court considers attacks on medical personnel and facilities as war crimes.^[24]^ We call upon the international community to protect HCWs in the Gaza strip.

### 4.1. Limitations of study

Our study has limitations. The study was initially planned to conclude data collection over 3 months; however, it took 9 months. The reason behind that is that HCWs were overworked during the study period, and the study included sensitive questions, like questions regarding the loss of family members. These 2 factors deterred many from completing the questionnaire. Moreover, the intermittent internet coverage and recurrent displacement of the research team slowed down the distribution of the survey. Another limitation is the sample size; our initial goal was to have more than 380 responses, but because of the slow process, investigators decided to conclude the study after we received more than 350 responses. We do not believe that the lower sample size affects the internal or external validity of the study because our primary goal was to measure and demonstrate the significant psychological stress in HCWs in Gaza, which we believe we were able to highlight.

## 5. Conclusion

In summary, our study highlights the immense psychological stress experienced by HCWs in the Gaza strip, driven by the long working hours, large influx of patients, and constant fear of injury or death. We hope that this study serves as compelling evidence and a reason to increase international healthcare missions, protect HCWs from military attacks, and, most importantly, a permanent cease-fire to preserve the lives of HCWs and civilians in the Gaza Strip.

## Acknowledgments

The authors dedicate this work to the memory of all healthcare providers who are detained or lost their lives during this war.

## Author contributions

**Conceptualization:** Abdallah Y. Naser, Moaath Mustafa Ali.

**Data curation:** Abdallah Y. Naser.

**Formal analysis:** Abdallah Y. Naser.

**Funding acquisition:** Abdallah Y. Naser, Moaath Mustafa Ali.

**Investigation:** Abdallah Y. Naser, Moaath Mustafa Ali, Abd Al-Karim H Sammour, Adnan N. Skaik, Ahmed Abed, Ruba Musallam, Muath Alsarafandi, Mohammed I. Halimy, Shadi Aljerjawi, Ahmed E. Habboub, Mohamad A. Rassoul, Abdullah Ghali, Mahmoud Loubani, Ruth-Kari Ramleth, Bilal Irfan, Farashin Silevany, Majdi S. Hamarshi, Amer Hamad Issa Abukhalaf, Bashar N Alzghoul.

**Methodology:** Abdallah Y. Naser, Moaath Mustafa Ali.

**Project administration:** Moaath Mustafa Ali, Bashar N. Alzghoul.

**Resources:** Abdallah Y. Naser, Moaath Mustafa Ali, Abd Al-Karim H Sammour, Adnan N. Skaik, Ahmed Abed, Ruba Musallam, Muath Alsarafandi, Mohammed I. Halimy, Shadi Aljerjawi, Ahmed E. Habboub, Mohamad A. Rassoul, Abdullah Ghali, Mahmoud Loubani, Ruth-Kari Ramleth, Bilal Irfan, Farashin Silevany, Majdi S. Hamarshi, Amer Hamad Issa Abukhalaf, Bashar N Alzghoul.

**Software:** Abdallah Y. Naser.

**Supervision:** Moaath Mustafa Ali.

**Validation:** Abdallah Y. Naser, Moaath Mustafa Ali, Bashar N Alzghoul.

**Visualization:** Abdallah Y. Naser, Moaath Mustafa Ali.

**Writing – original draft:** Abdallah Y. Naser, Moaath Mustafa Ali.

**Writing – review & editing:** Abdallah Y. Naser, Moaath Mustafa Ali, Abd Al-Karim H. Sammour, Adnan N. Skaik, Ahmed Abed, Ruba Musallam, Muath Alsarafandi, Mohammed I. Halimy, Shadi Aljerjawi, Ahmed E. Habboub, Mohamad A. Rassoul, Abdullah Ghali, Mahmoud Loubani, Ruth-Kari Ramleth, Bilal Irfan, Farashin Silevany, Majdi S. Hamarshi, Amer Hamad Issa Abukhalaf, Bashar N Alzghoul.

## Supplementary Material


