# Supplementary material for: Prevalence of stress, anxiety and depression among healthcare workers in the Gaza strip
Source: Medicine (Baltimore). 2025 Sep 5;104(36):e44195. doi: 10.1097/MD.0000000000044195 (PMC12419357; doi:10.1097/MD.0000000000044195)
Supplement: Supplementary file 1 [file medi-104-e44195-s001.pdf]

Supplementary material 1: English Version for Questionnaire

Start of Block: Default Question Block

This survey aims to understand the stress level experienced by healthcare workers who have provided healthcare in Gaza since 7 October 2023. It will help highlight the stress experienced by healthcare workers. The study was approved by the IRB of the Faculty of Pharmacy, Isra University. Investigators: Abdallah Y. Naser, PhD Department of Applied Pharmaceutical Sciences and Clinical Pharmacy Faculty of Pharmacy, Isra University Amman, Jordan Bashar Alzghoul, MD Pulmonary and Critical Care Division, University of Florida Gainesville, FL 32608 Moaath Mustafa Ali, MD, MPH Cleveland Clinic Taussig Cancer Center Cleveland, OH 44106

This survey is designed for anyone working in healthcare aged 18 years and above who worked in Gaza between 7 October 2023 and this date. Do you meet the criteria?

- No
- Yes

Skip To: End of Survey If This survey is designed for anyone working in healthcare aged 18 years and above who worked in Ga... = No

---

Sex:

- Female
- Male

Age Group:

18-23 years

24-30 years

31-40 years

41-50 years

51-60 years

61 years and above

What is your profession?

Nurse

Community Healthcare Workers

Doctor (i.e physician)

Support staff (janitor, food service staff, administrators, etc.)

EMT/Paramedic

Physician assistant or nurse practitione

Pharmacist

Other areas of patient care (laboratory technician, x-ray, therapist, front desk, etc.)

Others ____

Emergency medicine

Display this question:

If What is your profession? = Doctor (i.e physician)

Doctor's specialty?

- General Practitioner
- General Surgery
- Pediatrics
- Internal Medicine (general)
- Cardiology
- Neurology
- Dermatology
- Urology or Nephrology
- Hematology
- Obstetrics and Gynecology
- Orthopedics
- Dentistry
- Specialty Surgeon (e.g., ophthalmology, otolaryngology …etc.)
- Radiologist
- Anesthesiology
- Another specialty ____

Your main place of residence (before the war began)?

- Bani Suheila
- Beit Hanoun
- Beit Lahiya
- Deir Al-Balah
- Gaza City
- Jabalia
- Khan Yunis
- Rafah
- Another area, including outside the Gaza strip (mention it) ____

If you normally reside outside Gaza, have you come to Gaza to volunteer?

- No
- Yes
- Not applicable

If you normally reside outside Gaza, when did you start volunteering?

- Since the beginning of the war
- a month ago
- two months ago
- three months ago or more
- Not applicable

If you normally reside outside Gaza, will you volunteer again if you can?

- No
- Yes
- Not applicable

If you normally reside outside Gaza, do you encourage others to volunteer?

- No
- Yes
- Not applicable

If you normally reside outside Gaza, what is your main reason for volunteering?

- [ ] Humanitarian motivation
- [ ] Religious motivation
- [ ] Career motivation
- [ ] Another reason ____
- [ ] Not applicable

Where do you currently live?

Bani Suheila
Beit Hanoun
Beit Lahiya
Deir Al-Balah
Gaza City
Jabalia
Khan Yunis
Rafah
Another area (mention it) ____

How many times have you relocated since October 7, 2023?

Once
2-3 times
More than 3 times
No, I didn't move.

Where do you currently work?

Established Hospital (governmental)

Outpatient clinic

Field Hospital

Blood Bank

Dialysis center, separate from hospital

Somewhere else ____

Have you had to transfer your job from one location to another as a healthcare provider since October 7, 2023?

No

Yes

How many times have you had to transfer your job from one location to another as a healthcare provider since October 7, 2023?

No, I didn't have to change my job location

One time

2-3 times

More than 3 times

Are you currently making an income from the healthcare service you are providing in Gaza?

No

Yes

What is your longest continuous period of work (i.e., working shift) since October 7, 2023?

- Less than a week
- 7-30 days
- 31-60 days
- 61-90 days
- 91-120 days
- More than 120 days

On average, how many hours of sleep did you have per night over the past month?

- Less than two hours
- 2-4 hours
- 4-6 hours
- 6-8 hours
- More than 8 hours

Do you take a break from work every day?

- No
- Yes

Display this question:

If Do you take a break from work every day? = Yes

If yes, how long approximately?

- 2 hours
- 4 hours
- 6 hours
- 8 hours
- 10 hours
- More than 10 hours

Do you take a break from work every week?

No

Yes

Display this question:

If Do you take a break from work every week? = Yes

If yes, how long?

Less than a day

One day

Two days

More than two days

Have any of your healthcare colleagues you work(ed) with died because of military action since October 7, 2023?

No

Yes

Have any of your healthcare colleagues you work(ed) with been injured because of military action since October 7, 2023?

No

Yes

Have you been directly or indirectly targeted by military action since October 7, 2023?

No

Yes

Display this question:

If Have you been directly or indirectly targeted by military action since October 7, 2023? = Yes

If yes, you have been directly or indirectly targeted by military action since October 7, 2023, have you been injured?

No

Yes

Display this question:

If If yes, you have been directly or indirectly targeted by military action since October 7, 2023, h... = Yes

If yes, you have been directly or indirectly targeted by military action since October 7, 2023, have you been subjected to amputations?

No

Yes

Display this question:

If Have you been directly or indirectly targeted by military action since October 7, 2023? = Yes

If yes, did it happen while you were working?

No

Yes

Were any of the medical facilities where you worked involved in a military action?

No

Yes

Display this question:

If Were any of the medical facilities where you worked involved in a military action? = Yes

If yes, has any patient been injured?

No

Yes

Display this question:

If If yes, has any patient been injured? = Yes

Did any of the patients you were caring for die directly or indirectly from military actions?

No

Yes

Do you think the healthcare community outside Gaza has done enough to provide medical care in Gaza?

No

Yes

Are you currently food insecure?

No

Yes

Display this question:

If Are you currently food insecure? = Yes

How much weight have you lost in kilograms since October 7, 2023? (N.B. One pound equals 0.45 kg.)

I did not lose weight.

Less than 5 kg

6-10 kg

11-15 kg

More than 15 kg

I have lost weight, but I am not sure what weight I have lost.

Have you lost your primary home (i.e. partially or completely destroyed) since October 7, 2023?

No

Yes

Have you lost immediate family members (father, mother, brother, sisters, wife, or children) (i.e. died) since October 7, 2023?

No

Yes

Display this question:

If Have you lost immediate family members (father, mother, brother, sisters, wife, or children) (i.e... = Yes

How many immediate family members (father, mother, brother, sisters, wife, or children) have died/ been martyred?

0

1-2

3-4

5-6

7-8

More than 8

Have you lost any of your indirect family members (i.e. died) since October 7, 2023?

No

Yes

Display this question:

If Have you lost any of your indirect family members (i.e. died) since October 7, 2023? = Yes

How many indirect family members have died/been martyred?

0

1-2

3-4

5-6

7-8

More than 8

Do you have immediate family members who have been directly or indirectly targeted by military action since October 7, 2023?

No

Yes

Display this question:

If Do you have immediate family members who have been directly or indirectly targeted by military ac... = Yes

If yes, has anyone been injured?

No

Yes

Display this question:

If If yes, has anyone been injured? = Yes

If yes, have any amputations been performed?

No

Yes

Display this question:

If If yes, have any amputations been performed? = Yes

If yes, did anyone die?

No

Yes

Do you suffer from chronic medical disease(s)?

No

Yes

Display this question:

If Do you suffer from chronic medical disease(s)? = Yes

Have you been able to secure medications for these diseases you have?

No

Yes

Display this question:

If Do you suffer from chronic medical disease(s)? = Yes

Have you been able to receive health care for these diseases you have?

No

Yes

Over the past two weeks, how many times have you experienced any of the following problems?

	Not at all	Several days	More than half the days	Nearly every day
Little interest or pleasure in doing things?				
Feeling down, depressed, or hopeless?				
Trouble falling or staying asleep, or sleeping too much?				
Feeling tired or having little energy?				
Poor appetite or overeating?				
Feeling bad about yourself - or that you are a failure or have let yourself or your family down?				
Trouble concentrating on things, such as reading the newspaper or watching television?				
Moving or speaking so slowly that other people could have noticed? Or the opposite - being so fidgety or restless that you have been moving around a lot more than usual?				
Thoughts that you would be better off dead, or of hurting yourself in some way?				

Feeling nervous, anxious, or on edge
Not being able to stop or control worrying
Worrying too much about different things
Trouble relaxing
Being so restless that it's hard to sit still
Becoming easily annoyed or irritable
Feeling afraid as if something awful might happen

If you point out any of the problems above, how difficult for you to do your job, take care of household chores, or get along with other people?

- There is no difficulty
- There are some difficulties
- There are severe difficulties
- There are very complex difficulties

The following questions inquire about your feelings and thoughts over the past month. Please answer all the questions.

	Never	Amost never	Sometimes	Fairly often	Very often
How often have you been upset because of something that happened unexpectedly?					
How often have you felt that you were unable to control the important things in your life?					
How often have you felt nervous and stressed?					
How often have you felt confident about your ability to handle your personal problems?					
How often have you felt that things were going your way?					
How often have you found that you could not cope with all the things that you had to do?					
How often have you been able to control irritations in your life?					
How often have you felt that you were on top of things?					

How often have you been angered because of things that happened that were outside of your control?
How often have you felt difficulties were piling up so high that you could not overcome them?

End of Block: Default Question Block

Supplementary material 2: Arabic Version for Questionnaire

في الصحية الرعاية قدموا الذين الصحية الرعاية مجال في العاملون منه يعاني الذي التوتر مستوى فهم إلى تهدف مسحية دراسة هذه الصحية الرعاية مجال في العاملون منه يعاني الذي النفسي الضغط على الضوء تسليط في تساعد وسوف .2023 أكتوبر 7 منذ غزة.

7 بين ما الفترة في غزة في وعمل فوق فما عامًا 18 بعمر الصحية الرعاية مجال في يعمل شخص لأي الاستطلاع هذا تصميم تم المعايير؟ عليك تنطبق هل .التاريخ هذا وحتى 2023 أكتوبر

- لا
- نعم

الجنس:

- انثى
- ذكر

العمرية الفئة:

- سنة 18-23
- سنة 24-30
- سنة 31-40
- سنة 41-50
- سنة 51-60

○ فوق فما 61

مهنتك؟ هي ما

○ (ه)ممرض

○ المجتمعية الصحية الرعاية مجال في العاملين

○ طبيب

○ الدعم موظفو (بواب، موظفو خدمات الطعام، الإداريون، إلخ.)

○ EMT/مسعف

○ ممارس ممرض أو طبيب مساعد

○ صيدلاني

○ مجالات رعاية المرضى الأخرى (فني المختبر، الأشعة السينية، المعالج، مكتب الاستقبال، وما إلى ذلك)

○ آخرون ____

○ طوارئ طبيب

الطبيب؟ تخصص

○ عام طبيب

○ عامة جراحة

○ اطفال

○ باطني طبيب

- قلب طبيب
- اعصاب طبيب
- جلدية طبيب
- كلى و بوليه مسالك طبيب
- دم امراض طبيب
- توليد و نسائية طبيب
- عظام طبيب
- اسنان طبيب
- متخصصة جراحه طبيب
- تشخيصية اشعة طبيب
- تخدير طبيب
- اخر تخصص ______________________________

؟(الحرب بدء قبل) الاساسي سكنك مكان

- سهيلا بني
- حانون بيت
- لاهيا بيت
- البلح دير
- غزة
- جباليا

- ◯ يونس خان
- ◯ رفح
- ◯ (اذكرها) اخرى منطقة ____

للتطوع؟ غزة إلى أتيت هل ،غزة خارج كنت إذا

- ◯ لا
- ◯ نعم
- ◯ ينطبق لا

التطوعي العمل بدأت متى غزة خارج كنت إذا

- ◯ الحرب بداية منذ
- ◯ شهر منذ
- ◯ شهرين منذ
- ◯ شهور ثلاثة منذ
- ◯ ينطبق لا

استطعت؟ إذا أخرى مرة ستتطوع هل ،غزة خارج كنت إذا

- ○ لا
- ○ نعم
- ○ ينطبق لا

التطوع؟ على الآخرين تشجع هل ،غزة خارج كنت إذا

- ○ لا
- ○ نعم
- ○ ينطبق لا

للتطوع؟ دفعك الذي الرئيسي السبب هو ما ،غزة خارج كنت إذا

- ☐ انساني دافع
- ☐ ديني دافع
- ☐ مهني دافع
- ☐ اخر سبب ____
- ☐ ينطبق لا

الحالي؟ سكنك مكان

- ○ سهيلا بني

- ○ حانون بيت
- ○ لاهيا بيت
- ○ البلح دير
- ○ غزة
- ○ جباليا
- ○ يونس خان
- ○ رفح
- ○ (اذكرها) اخرى منطقة ____

؟2023 أكتوبر 7 منذ بالانتقال قمت مرة كم

- ○ مرة
- ○ مرات 3-2
- ○ مرات 3 من اكثر
- ○ انتقل لم لا

حالياً؟ تعمل اين

- ○ جكومي مستشفى
- ○ خارجية عيادة
- ○ ميداني مستشفى

- الدم بنك
- المستشفى خارج الكلى غسيل مركز
- اخر مكان ____

؟ 2023 أكتوبر 7 منذ صحية رعاية كمقدم وظيفتك نقل إلى اضطررت هل

- لا
- نعم

؟ 2023 أكتوبر 7 منذ صحية رعاية كمقدم وظيفتك نقل إلى اضطررت مرة كم

- وظيفتي مكان لتغيير اضطر لم لا
- واحدة مرة
- مرات 3-2
- مرات 3 من اكثر

العمل؟ هذا من دخلا حاليا تحقق هل

- لا
- نعم

؟ 2023 أكتوبر 7 منذ لك متواصلة عمل فترة أطول هي ما

- ○ اسبوع من اقل
- ○ يوم 30 الى ايام 7
- ○ يوم 60 الى يوم 31
- ○ يوم 90 الى يوم 61
- ○ يوم 120 الى يوم 91
- ○ يوم 120 من اكثر

الماضي؟ الشهر خلال ليلة كل نمتها التي الساعات عدد كم ،المتوسط في

- ○ ساعتين من اقل
- ○ ساعات 2-4
- ○ ساعات 4-6
- ○ ساعات 6-8
- ○ ساعات 8 من اكثر

العمل؟ من يوم كل استراحة تأخذ هل

- ○ لا

- نعم

الوقت؟ من كم ،بنعم الإجابة كانت إذا

- ساعتان
- ساعات 4
- ساعات 6
- ساعات 8
- ساعات 10
- ساعات 10 من اكثر

العمل؟ من أسبوع كل استراحة تأخذ هل

- لا
- نعم

الوقت؟ من كم ،بنعم الإجابة كانت إذا

- يوم من اقل
- يوم
- يومين
- يومين من اكثر

؟ 2023 أكتوبر 7 منذ عسكري عمل نتيجة معهم تعمل الذين الصحية الرعاية مجال في زملائك من أي توفي هل

- لا
- نعم

؟ 2023 أكتوبر 7 منذ العسكري العمل بسبب معهم تعمل الذين الصحية الرعاية مجال في زملائك من أي أصيب هل

- لا
- نعم

؟ 2023 أكتوبر 7 منذ عسكري بعمل مباشر غير أو مباشر بشكل استهدافكم تم هل

- لا
- نعم

اصبت؟ هل, 2023 أكتوبر 7 منذ عسكري بعمل مباشر غير أو مباشر بشكل استهدافكم تم نعم اذا

- لا
- نعم

البتر؟ لعمليات تعرضت هل ، 2023 أكتوبر 7 منذ عسكري بعمل مباشر غير أو مباشر بشكل استهدافكم تم بنعم الإجابة كانت إذا

○ لا

○ نعم

عملك؟ أثناء ذلك حدث فهل ،بنعم الإجابة كانت إذا

○ لا

○ نعم

العسكري؟ بالعمل بها عملت التي الطبية المرافق من أي استهداف تم هل

○ لا

○ نعم

مريض؟ أي أصيب هل ،بنعم الإجابة كانت إذا

○ لا

○ نعم

مباشر؟ غير أو مباشر بشكل العسكرية الأعمال بسبب بهم تعتني كنت الذين مرضاك من أي توفي هل

○ لا

○ نعم

هل تعتقد أن مجتمع الرعاية الصحية خارج غزة قام بما يكفي لتوفير الرعاية الطبية في غزة؟

- لا
- نعم

هل تعاني حاليا من انعدام الأمن الغذائي؟

- لا
- نعم

ما مقدار الوزن الذي فقدته بالكيلوجرام منذ 7 أكتوبر 2023؟

- لم افقد وزن
- اقل من 5 كيلو غرام
- 6-10 كيلو غرام
- 11-15 كيلو غرام
- اكثر من 15 كيلو غرام
- فقدت وزناً لكني غير متأكد من الوزن الذي فقدته

هل فقدت مسكنك الأساسي (أي المنزل المدمر جزئيًا أو كليًا) منذ 7 أكتوبر 2023 ؟

- ◯ لا
- ◯ نعم

هل فقدت فرد من أفراد عائلتك المباشرين (الأب والأم والأخ والأخوات والزوجة والأطفال) منذ 7 أكتوبر 2023 ؟

- ◯ لا
- ◯ نعم

كم فرد من افراد العائلة المباشرين (الأب والأم والأخ والأخوات والزوجة والأطفال) فقدت (استشهد)؟

- ◯ 0
- ◯ 1-2
- ◯ 3-4
- ◯ 5-6
- ◯ 7-8
- ◯ اكثر من 8

هل فقدت فرد من أفراد عائلتك الغير المباشرين منذ 7 أكتوبر 2023 ؟

- ◯ لا
- ◯ نعم

كم فرد من افراد العائلة الغير المباشرين فقدت (استشهد)؟

- 0
- 1-2
- 3-4
- 5-6
- 7-8
- اكثر من 8

هل لديك أفراد من عائلتك المباشرين مستهدفون بشكل مباشر أو غير مباشر بعمل عسكري منذ 7 أكتوبر 2023 ؟

- لا
- نعم

إذا كانت الإجابة بنعم، فهل أصيب أحد؟

- لا
- نعم

إذا كانت الإجابة بنعم، هل تم إجراء أي عمليات بتر؟

- لا

نعم

أحد؟ مات هل ،بنعم الإجابة كانت إذا

لا

نعم

مزمنة؟ طبية (أمراض) مرض من تعاني هل

لا

نعم

الأدوية؟ تأمين من تمكنتم هل

لا

نعم

منها؟ تعاني التي الامراض لهذه الصحية الرعاية تأمين من تمكنت هل

لا

نعم

التالية؟ المشاكل من أي من عانيت مرة كم ،الماضيين الأسبوعين خلال

	ولا مرة	عدة ايام	أكثر من نصف الايام	تقريباً كل يوم
قلة أو الاهتمام قلة بممارسة الاستمتاع عمل بأي بالقيام	○	○	○	○
أو بالحزن الشعور اليأس أو الصدر ضيق	○	○	○	○
نوم أو النوم في صعوبة من أكثر النوم أو متقطع المعتاد	○	○	○	○
أو بالتعب الشعور من جداً القليل بامتلاك الطاقة	○	○	○	○
في الزيادة أو الشهية قلة المعتاد عن الطعام تناول	○	○	○	○
عن الرضا بعدم الشعور بأنك الشعور أو النفس أو نفسك أخذلت قد عائلتك	○	○	○	○
مثلاً التركيز في صعوبة أو الصحيفة قراءة أثناء التلفزيون مشاهدة	○	○	○	○
بطء أو الحركة في بطء هو عما التحدث في ملحوظة لدرجة معتاد على أو / الآخرين من التحدث ذلك من العكس الحركة وكثرة بسرعة المعتاد من أكثر	○	○	○	○
من بأنه أفكار راودتك أو ميتا آنت لو الأفضل بإيذاء تقوم بأن أفكار النفس	○	○	○	○
أو بالغضب الشعور الشديد الانفعال أو القلق.	○	○	○	○
إنهاء على القدرة عدم فيه التحكّم أو القلق.	○	○	○	○
أشياء على المفرط القلق مختلفة.	○	○	○	○

في الصعوبة الاسترخاء.	○	○	○	○
لدرجة الاضطراب شدة هدوء في البقاء صعوبة.	○	○	○	○
أو الانزعاج في السرعة الانفعال.	○	○	○	○
لو كما بالخوف الشّعور قد فضيعا شيئا أن يحدث.	○	○	○	○

مع الانسجام أو ،المنزلية بالأمور الاعتناء ،بعملك القيام المشاكل هذه عليك صعّبت درجة أية فإلى ،أعلاه المشاكل من أية إلى أشرت إذا آخرين؟ أشخاص

○ صعوبة اي هناك ليست

○ الصعوبات بعض هناك

○ شديدة صعوبات هناك

○ التعقيد بالغة صعوبات هناك

.. الأسئلة جميع على الاجابة منك نرجو .الماضي الشهر خلال أفكارك و مشاعرك عن تستفسر التالية الأسئلة

	(ابداً) اطلاقاً	نادراً	احياناً	كثيراً	اغلب في) دائماً (الاحيان
الشهر خلال أي الى ،الماضي أحسست مدى بسبب بالانزعاج غير أمر حدوث ؟ متوقع	○	○	○	○	○
الشهر خلال أي الى ،الماضي	○	○	○	○	○

بعدم أحسست مدى<br>التحكم على القدرة<br>الهامة الأمور في<br>؟ بحياتك					
الشهر خلال<br>أي الى ،الماضي<br>أحسست مدى<br>الضغط و بالتوتر<br>؟ النفسي	○	○	○	○	○
الشهر خلال<br>أي الى ،الماضي<br>بالثقة أحسست مدى<br>على قدرتك في<br>مشاكلك مع التعامل<br>؟ الخاصة	○	○	○	○	○
الشهر خلال<br>أي الى ،الماضي<br>أن أحسست مدى<br>كما تسير الأمور<br>؟ تريد	○	○	○	○	○
الشهر خلال<br>أي الى ،الماضي<br>نفسك وجدت مدى<br>على قادر غير<br>كل مع التأقلم<br>الواجب الأمور<br>؟ بها القيام عليك	○	○	○	○	○
الشهر خلال<br>أي إلى ،الماضي<br>من تمكنت مدى<br>الأمور في التحكم<br>؟ تزعجك التي	○	○	○	○	○
الشهر خلال<br>أي الى ،الماضي<br>بأنك أحسست مدى<br>) الأمور زمام تملك<br>كافة على مسيطر<br>؟ (أمورك	○	○	○	○	○
الشهر خلال<br>أي الى ،الماضي<br>أحسست مدى<br>بسبب بالغضب<br>عن خارجة أمور<br>؟ تحكمك	○	○	○	○	○

الشهر خلال<br>أي إلى ،الماضي<br>بأن أحسست مدى<br>تتراكم الصعاب<br>لم أنك لدرجة عليك<br>التغلب تستطيع تعد<br>؟ عليها	○	○	○	○	○

End of Block: Default Question Block

Supplementary material 3: Message used for survey dissemination, in English and Arabic

Dear Healthcare Providers,

We invite you to participate in a survey aimed at measuring the psychological stress experienced by healthcare workers amidst the ongoing conflict in Gaza, which began on October 7, 2023.

Eligibility:
This survey is open to all healthcare professionals, including nurses, pharmacists, paramedics, and other medical staff who have worked in Gaza since October 7, 2023. Please note that students are not eligible to participate.

Study Objective:
The primary goal of this study is to highlight the psychological stress faced by healthcare workers in Gaza. Our long-term objective is to use the results to advocate for increased medical missions to Gaza and improved support for healthcare professionals working in such challenging conditions.

Survey Details:

- The questionnaire contains no personal information and can be completed on any device, including a mobile phone, desktop, or laptop.
- The study has been approved by the Institutional Review Board (IRB) at the Faculty of Pharmacy, Isra University.

Investigators:

1. **Abdallah Y. Naser, PhD**
   Department of Applied Pharmaceutical Sciences and Clinical Pharmacy, Faculty of Pharmacy, Isra University, Amman, Jordan
2. **Bashar Alzghoul, MD**
   Pulmonary and Critical Care Division, University of Florida, Gainesville, FL, USA
3. **Moaath Mustafa Ali, MD, MPH**
   Cleveland Clinic Taussig Cancer Center, Cleveland, OH, USA

English Survey Link: https://corexmsj94w3kz8bjy6m.qualtrics.com/jfe/form/SV_eRTLDHeySBaMjNY

Arabic Survey Link: https://corexmsj94w3kz8bjy6m.qualtrics.com/jfe/form/SV_0ocHiaqgYow3uBg

Thank you for your valuable time and contribution to this important research.

Moaath Mustafa Ali, MD, MPH

Message used for survey dissemination, in Arabic

وبركاته الله ورحمة عليكم السلام:

غزة على الحرب خضم في الطبية الكوادر لدى والتوتر العمل عن الناتج النفسي الضغط مقدار لقياس يهدف البحثي الاستبيان هذا ☆
. اكتوبر من السابع منذ

داخل النظافة ووعمال والإسعاف والصيادلة والمرضين الاطباء وشمل ،الصحي القطاع في يعمل من لجميع الاستبيان هذا ☆
.التقنيين وكذلك المستشفى

الوفود من والزائرين غزة في الطبي القطاع في العاملين معاناة لتوثيق الاستبيان يهدف .شخصية معلومات أي يحتوي لا الاستبيان ☆
.علمية بطريقة

☜ ،.الإلكتروني والبريد المسجات طريق عن الرسالة هذه مع الاستبيان هذا توزيع يمكن

.أردنيين اطباء من الدراسة تصميم تم

١. صيدلة دكتور ،ناصر عبدلله دكتور

٢. الحثيثة والعناية تنفسية اخصائي ،زغلول بشار دكتور

٣. والسرطان الدم أمراض اخصائي ،علي مصطفى معاذ دكتور

☜ لتعبئته التالي الاستبيان رابط على اضغط:

https://corexmsj94w3kz8bjy6m.qualtrics.com/jfe/form/SV_0ocHiaqgYow3uBg
