# Supplementary material for: Prevalence of stress, anxiety and depression among healthcare workers in the Gaza strip
Source: Medicine (Baltimore). 2025 Sep 5;104(36):e44195. doi: 10.1097/MD.0000000000044195 (PMC12419357; doi:10.1097/MD.0000000000044195)
Supplement: Supplementary file 2 [file medi-104-e44195-s002.docx]

Supplementary Table S1. Linear regression analysis of factors predicting depression score measured by Patient Health Questionnaire-9 (PHQ-9).

| Model |  | Unstandardized Coefficients | | P value | 95.0% CI for B | |
| --- | --- | --- | --- | --- | --- | --- |
|  |  | B | Std. Error |  | Lower Bound | Upper Bound |
|  | (Constant) | 0.855 | 3.066 | 0.781 | -5.224 | 6.934 |
|  | Stress score | 0.179 | 0.109 | 0.104 | -0.037 | 0.396 |
|  | Anxiety score | 0.836 | 0.086 | 0.000 | 0.666 | 1.006 |
| Gender | Female | Reference | | | | |
|  | Male | -0.427 | 1.120 | 0.704 | -2.648 | 1.793 |
| Age | 18-23 | Reference | | | | |
|  | 24-30 | 1.574 | 1.470 | 0.287 | -1.340 | 4.488 |
|  | 31-40 | -0.628 | 1.767 | 0.723 | -4.131 | 2.875 |
|  | 41-50 | -1.099 | 2.098 | 0.601 | -5.258 | 3.059 |
|  | 51-60 | -3.300 | 2.204 | 0.137 | -7.671 | 1.070 |
|  | 61- | -2.386 | 2.740 | 0.386 | -7.819 | 3.047 |
| Residency | Inside Gaza | Reference | | | | |
|  | Outside Gaza | 1.076 | 3.233 | 0.740 | -5.335 | 7.486 |
| Practice | General practitioner | Reference | | | | |
|  | General surgery | 1.787 | 2.828 | 0.529 | -3.820 | 7.394 |
|  | Pediatrics | 3.365 | 2.537 | 0.188 | -1.664 | 8.395 |
|  | Internal medicine | -1.581 | 4.666 | 0.735 | -10.831 | 7.670 |
|  | Dermatology | 0.776 | 3.537 | 0.827 | -6.237 | 7.788 |
|  | Urology and nephrology | -0.072 | 4.880 | 0.988 | -9.748 | 9.604 |
|  | Obstetrics and gynecology | 2.715 | 1.192 | 0.025 | 0.351 | 5.078 |
|  | Orthopedics | 2.991 | 4.432 | 0.501 | -5.795 | 11.778 |
|  | Dentist | 1.471 | 1.901 | 0.441 | -2.298 | 5.241 |
|  | Special surgeon | 1.277 | 1.499 | 0.396 | -1.695 | 4.249 |
|  | Radiologist | 3.557 | 2.695 | 0.190 | -1.785 | 8.899 |
|  | Another specialty | 0.365 | 2.183 | 0.868 | -3.964 | 4.694 |

Supplementary Table S2. Linear regression analysis of factors predicting anxiety score measured by the Generalized Anxiety Disorder 7 (GAD-7) scale.

| Model |  | Unstandardized Coefficients | | P value | 95.0% CI for B | |
| --- | --- | --- | --- | --- | --- | --- |
|  |  | B | Std. Error |  | Lower Bound | Upper Bound |
|  | (Constant) | -2.098 | 2.515 | 0.406 | -7.084 | 2.888 |
|  | Stress score | 0.270 | 0.087 | 0.003 | 0.097 | 0.442 |
|  | Depression score | 0.566 | 0.058 | 0.000 | 0.451 | 0.681 |
| Gender | Female | Reference | | | | |
|  | Male | -0.063 | 0.922 | 0.945 | -1.891 | 1.764 |
| Age | 18-23 | Reference | | | | |
|  | 24-30 | -0.327 | 1.215 | 0.788 | -2.736 | 2.082 |
|  | 31-40 | 0.440 | 1.454 | 0.763 | -2.442 | 3.322 |
|  | 41-50 | -0.437 | 1.727 | 0.801 | -3.861 | 2.987 |
|  | 51-60 | 0.589 | 1.831 | 0.748 | -3.041 | 4.220 |
|  | 61- | -2.922 | 2.244 | 0.196 | -7.372 | 1.527 |
| Residency | Inside Gaza | Reference | | | | |
|  | Outside Gaza | -1.108 | 2.659 | 0.678 | -6.379 | 4.164 |
| Practice | General practitioner | Reference | | | | |
|  | General surgery | 1.344 | 2.327 | 0.565 | -3.270 | 5.957 |
|  | Pediatrics | 1.949 | 2.095 | 0.354 | -2.205 | 6.104 |
|  | Internal medicine | 5.514 | 3.803 | 0.150 | -2.025 | 13.053 |
|  | Dermatology | -2.421 | 2.901 | 0.406 | -8.172 | 3.330 |
|  | Urology and nephrology | 2.412 | 4.008 | 0.549 | -5.534 | 10.358 |
|  | Obstetrics and gynecology | -1.060 | 0.999 | 0.291 | -3.041 | 0.921 |
|  | Orthopedics | -4.867 | 3.623 | 0.182 | -12.049 | 2.315 |
|  | Dentist | 0.356 | 1.568 | 0.821 | -2.753 | 3.465 |
|  | Special surgeon | -0.096 | 1.237 | 0.938 | -2.549 | 2.357 |
|  | Radiologist | -1.060 | 2.232 | 0.636 | -5.486 | 3.366 |
|  | Another specialty | -0.483 | 1.796 | 0.789 | -4.043 | 3.077 |

Supplementary Table S3. Linear regression analysis of factors predicting Stress score measured by Perceived Stress Scale (PSS-10)

| Model |  | Unstandardized Coefficients | | P value | 95.0% CI for B | |
| --- | --- | --- | --- | --- | --- | --- |
|  |  | B | Std. Error |  | Lower Bound | Upper Bound |
|  | (Constant) | 20.937 | 1.763 | 0.000 | 17.442 | 24.432 |
|  | Depression score | 0.138 | 0.084 | 0.104 | -0.029 | 0.305 |
|  | Anxiety score | 0.307 | 0.099 | 0.003 | 0.110 | 0.503 |
| Gender | Female | Reference | | | | |
|  | Male | 0.046 | 0.983 | 0.962 | -1.903 | 1.996 |
| Age | 18-23 | Reference | | | | |
|  | 24-30 | -1.578 | 1.287 | 0.223 | -4.130 | 0.975 |
|  | 31-40 | -0.863 | 1.549 | 0.578 | -3.934 | 2.207 |
|  | 41-50 | -1.928 | 1.833 | 0.295 | -5.562 | 1.707 |
|  | 51-60 | 0.158 | 1.954 | 0.936 | -3.716 | 4.033 |
|  | 61- | 0.860 | 2.411 | 0.722 | -3.921 | 5.641 |
| Residency | Inside Gaza | Reference | | | | |
|  | Outside Gaza | 2.869 | 2.825 | 0.312 | -2.731 | 8.469 |
| Practice | General practitioner | Reference | | | | |
|  | General surgery | 0.643 | 2.485 | 0.796 | -4.284 | 5.571 |
|  | Pediatrics | -3.413 | 2.220 | 0.127 | -7.814 | 0.987 |
|  | Internal medicine | -5.611 | 4.060 | 0.170 | -13.659 | 2.438 |
|  | Dermatology | 4.569 | 3.072 | 0.140 | -1.522 | 10.660 |
|  | Urology and nephrology | 8.660 | 4.199 | 0.042 | 0.336 | 16.985 |
|  | Obstetrics and gynecology | 0.625 | 1.070 | 0.560 | -1.496 | 2.746 |
|  | Orthopedics | 0.487 | 3.896 | 0.901 | -7.237 | 8.212 |
|  | Dentist | 0.877 | 1.671 | 0.601 | -2.435 | 4.189 |
|  | Special surgeon | -0.824 | 1.317 | 0.533 | -3.436 | 1.788 |
|  | Radiologist | 1.243 | 2.380 | 0.603 | -3.476 | 5.962 |
|  | Another specialty | 4.771 | 1.859 | 0.012 | 1.086 | 8.457 |
